# Baseline Inflammatory and Nutritional Indices Show Limited Discrimination for Major Pathological Response and Survival After Neoadjuvant Chemotherapy for Gastric Cancer: A Retrospective Comparison of Eleven Indices

**DOI:** 10.3390/jcm15176893

**Published:** 2026-09-06

**Authors:** Erkut Demirciler, Kübra Canaslan, Halil İbrahim Ellez, Seval Akay, Gökalp Okut, Serhan Derici, Asuman Argon, Anıl Aysal Ağalar, Hüseyin Salih Semiz

**Affiliations:** 1 Department of Medical Oncology, Izmir City Hospital, Şevket İnce District, 2148/11th Street No:1/11, Izmir 35540, Turkey; drsevalsekerler@gmail.com; 2 Department of Medical Oncology, Sanliurfa Mehmet Akif Inan Education and Research Hospital, Sanliurfa 63040, Turkey; kubracanaslan@gmail.com (K.C.); h.ibrahimellez@gmail.com (H.İ.E.); 3 Department of General Surgery, Izmir City Hospital, Izmir 35540, Turkey; gokalp.okut@gmail.com; 4 Department of General Surgery, Faculty of Medicine, Dokuz Eylül University, Izmir 35330, Turkey; dr.serhan.derici@gmail.com; 5 Department of Pathology, Izmir City Hospital, Izmir 35330, Turkey; asumanargon@gmail.com; 6 Department of Pathology, Faculty of Medicine, Dokuz Eylül University, Izmir 35330, Turkey; anilaysalagalar@gmail.com; 7 Institute of Oncology, Dokuz Eylül University, Izmir 35330, Turkey; hsalihsemiz@hotmail.com

**Keywords:** gastric cancer, neoadjuvant chemotherapy, systemic inflammatory markers, Prognostic Nutritional Index, platelet-to-lymphocyte ratio, modified Glasgow Prognostic Score, Mandard tumor regression grade

## Abstract

**Background/Objectives:** While perioperative chemotherapy is standard for locally advanced gastric cancer, no baseline blood marker reliably identifies which patients will respond to treatment. In this retrospective study, we compared eleven inflammatory and nutritional indices against pathological response and survival using data collected between 2020 and 2025. **Methods:** We analyzed 130 patients with gastric adenocarcinoma receiving perioperative systemic therapy at two centers. Eleven indices—the neutrophil-to-lymphocyte ratio (NLR), systemic immune–inflammation index (SII), Prognostic Nutritional Index (PNI), and eight further indices defined—were assessed against the Mandard tumor regression grade (TRG), overall survival (OS), and disease-free survival (DFS). **Results:** The median age was 63 years, and the median follow-up was 22.1 months from diagnosis. A Mandard TRG was assigned in 123 patients, 32 of whom achieved a major pathological response (TRG 1–2). The PNI had the highest point estimate of discrimination for major pathological response (AUC 0.618, 95% CI 0.490–0.746; *p* = 0.070), but its area under the curve did not differ significantly from that of any other index (all *p* ≥ 0.18), and bootstrap validation incorporating index selection and threshold derivation yielded an optimism-corrected AUC of 0.567. While PNI ≥ 53 was nominally associated with TRG 1–2 (OR 3.823, 95% CI 1.503–9.722; *p* = 0.005), no association survived correction for multiple comparisons, and the PNI was selected as the best-performing index in only 49% of bootstrap resamples. No index showed a statistically significant association with overall survival, and the event count is insufficient to establish the presence or absence of an association. **Conclusions:** None of the eleven baseline blood-based indices demonstrated statistically robust or clinically useful discrimination for major pathological response, and none were associated with survival. Although the PNI represented the most promising exploratory signal, its apparent advantage was not statistically distinguishable from the other indices and did not survive internal validation. The threshold of approximately 53 should be regarded as hypothesis-generating only.

## 1. Introduction

Gastric cancer is the fifth most common malignancy globally and the fourth leading cause of cancer-related mortality, with approximately 968,000 new cases and 660,000 deaths annually [1]. Although localized disease is associated with a relatively favorable prognosis, patients with regional lymph node involvement have a five-year survival rate of only 37%, highlighting the critical need for effective multimodal treatment strategies [2]. The landmark MAGIC trial transformed perioperative management by showing that adding epirubicin, cisplatin, and fluorouracil to surgery significantly improves survival in resectable gastric and gastro-esophageal junction (GEJ) cancer [3]. Subsequently, the FLOT4-AIO trial established FLOT (5-fluorouracil, leucovorin, oxaliplatin, and docetaxel) as the standard of care, surpassing ECF/ECX-based regimens and extending median overall survival beyond four years in locally advanced cases [4]. More recently, the MATTERHORN trial showed that adding the programmed death ligand-1 (PD-L1) inhibitor durvalumab to perioperative FLOT improves pathological complete response and event-free survival [5,6]. However, the substantial cost of immunotherapy remains a major barrier for healthcare systems and patients, underscoring the need for careful patient selection [7].

Systemic inflammatory and nutritional indices—calculated from routine blood counts and serum biochemistry—have therefore emerged as practical, low-cost surrogates for the host immune microenvironment and systemic inflammatory status. Their prognostic utility is grounded in the well-established role of cancer-related inflammation in facilitating tumor immune evasion, promoting angiogenesis, and enabling metastatic dissemination [8]. Large-scale meta-analyses have since established the prognostic value of indices such as the neutrophil-to-lymphocyte ratio (NLR) and the Prognostic Nutritional Index (PNI)—the latter originally developed to predict postoperative complications in malnourished surgical patients [9,10]—across multiple gastrointestinal malignancies [11].

Although the prognostic significance of individual inflammatory markers is well established, their comparative performance in the perioperative chemotherapy context for gastric cancer remains insufficiently characterized [12]. Notably, few studies have concurrently assessed a comprehensive panel of eleven composite indices—including recently developed scores such as the systemic immune–inflammation index (SII), systemic inflammatory response index (SIRI), hemoglobin–albumin–lymphocyte–platelet (HALP) score, albumin–bilirubin index (ALBI), platelet–albumin–bilirubin index (PALBI), platelet-to-lymphocyte ratio (PLR), and modified Glasgow Prognostic Score (mGPS)—against pathological tumor regression and long-term survival in the neoadjuvant setting [12,13]. We restricted the panel to indices calculable from a standard complete blood count and routine biochemistry so that any center could reproduce them without additional testing; alongside the ratio-based scores, we included the PLR and mGPS, both of which have a larger validation base in gastrointestinal cancer than several newer composite indices. In this study, to address this gap, we systematically evaluated these indices in a real-world gastric cancer cohort to identify the blood-based biomarker most reliably associated with the Mandard tumor regression grade (TRG) and clinical outcomes.

## 2. Materials and Methods

### 2.1. Study Design and Population

In this retrospective study, we collected patient data from two oncology centers in Izmir, Turkey—Izmir City Hospital and Dokuz Eylül University Faculty of Medicine—between 2020 and 2025. Patients were eligible if they met all of the following criteria: age ≥ 18 years; histopathologically confirmed gastric or gastro-esophageal junction (GEJ) adenocarcinoma; locally advanced, resectable disease (cT2–T4 and/or cN+, M0) confirmed by preoperative computed tomography (CT) staging; case reviewed and approved for perioperative systemic therapy by the multidisciplinary tumor board; receipt of at least one cycle of neoadjuvant chemotherapy followed by curative-intent (R0) gastrectomy; availability of baseline laboratory values (complete blood count and serum biochemistry) obtained within seven days before the initiation of systemic therapy; and a complete postoperative histopathological report including the Mandard TRG, ypT stage, and ypN stage. We excluded patients with synchronous or metachronous second primary malignancies, distant metastasis (M1) at the time of diagnosis or before surgery, non-curative surgical intent, or insufficient baseline laboratory data to calculate at least one inflammatory index. As this was an observational retrospective study, we did not include a control arm; the study was designed to reflect real-world clinical outcomes in a middle-income healthcare setting.

**Figure 1:**
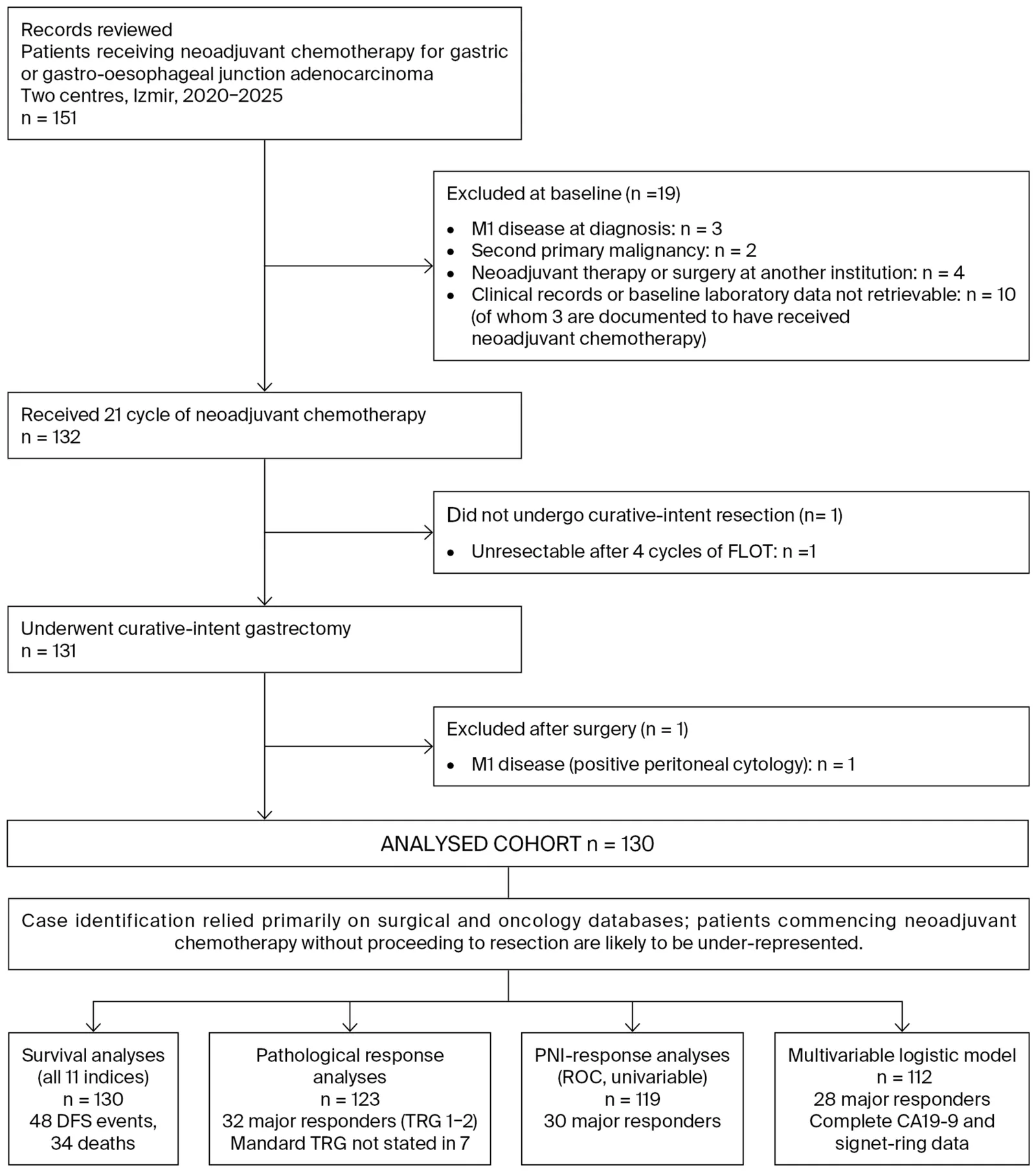
Flow of participants. The records of 151 patients who received neoadjuvant chemotherapy for locally advanced gastric or gastro-esophageal junction adenocarcinoma at the two participating centers between 2020 and 2025 were reviewed. We excluded 19 patients at baseline, leaving 132 who received at least one cycle of neoadjuvant chemotherapy. One became unresectable after four cycles of FLOT; of the 131 who underwent gastrectomy, one was excluded when peritoneal cytology proved positive, leaving 130 patients in the analyzed cohort. Because case identification relied primarily on the surgical and oncology databases of the two centers, patients who started neoadjuvant chemotherapy but did not proceed to resection are likely underrepresented. We assigned a Mandard tumor regression grade (TRG) in 123 patients, of whom 32 achieved a major pathological response (TRG 1–2); the pathology report did not state the grade for seven patients. The Prognostic Nutritional Index (PNI) was calculable in 126 patients, with serum albumin being unavailable in four; 119 had both an available PNI and an assigned TRG, of whom 30 achieved a major pathological response. The multivariable model, additionally adjusted for CA19-9 level and signet-ring cell component, included 112 patients with complete data, of whom 28 achieved a major pathological response. Denominators for individual indices vary with laboratory data availability and are reported in Supplementary Tables S1–S4.

The records of 151 patients who received neoadjuvant chemotherapy for gastric or gastro-esophageal junction adenocarcinoma at the two centers during the study period were reviewed. We excluded 19 patients at baseline: three had M1 disease at diagnosis; two had a second primary malignancy; four had received neoadjuvant therapy or undergone surgery at another institution; and ten had no retrievable clinical records or baseline laboratory data. This left 132 patients documented as having received at least one cycle of neoadjuvant chemotherapy. One did not proceed to resection, as the disease became unresectable after four cycles of FLOT. Of the 131 who underwent curative-intent gastrectomy, one was excluded when peritoneal cytology proved positive, leaving 130 patients in the analyzed cohort (Figure 1). Because case identification relied primarily on the surgical and oncology databases of the two centers, patients who started neoadjuvant chemotherapy without proceeding to resection are likely underrepresented, and the cohort should be understood as a surgery-completer population.

All patients underwent contrast-enhanced computed tomography of the thorax, abdomen, and pelvis for baseline staging, and all cases were reviewed by a multidisciplinary tumor board before treatment. We performed diagnostic laparoscopy selectively in patients with suspected serosal involvement, and endoscopic ultrasound was not routinely available; practice did not differ materially between the two centers. Staging investigations followed routine institutional practice rather than a study protocol, and staging extent was not uniform across the study period.

### 2.2. Data Sources and Variables

We extracted clinical data from electronic medical records and institutional databases. The collected variables included age; sex; tumor location; histologic subtype; tumor grade; biomarker status (EBV, MMR, HER2, p53); details of the perioperative treatment regimen (neoadjuvant and adjuvant regimens, number of cycles, dose modifications); surgical records (extent of lymphadenectomy, number of nodes harvested); and postoperative histopathologic features, including the Mandard TRG, ypT stage, and ypN stage.

The baseline laboratory values were defined as tests obtained within seven days before the initiation of systemic therapy for a pathologically confirmed diagnosis of gastric cancer. The following eleven inflammatory and nutritional indices were calculated from routine complete blood count and serum biochemistry parameters:

Neutrophil-to-lymphocyte ratio (NLR) = neutrophil count/lymphocyte count; lymphocyte-to-monocyte ratio (LMR) = lymphocyte count/monocyte count; neutrophil-to-monocyte ratio (NMR) = neutrophil count/monocyte count; systemic immune–inflammation index (SII) = (platelet count × neutrophil count)/lymphocyte count; systemic inflammatory response index (SIRI) = (monocyte count × neutrophil count)/lymphocyte count; Prognostic Nutritional Index (PNI) = (10 × serum albumin [g/dL]) + (0.005 × total lymphocyte count [/mm^3^]) [10]; hemoglobin–albumin–lymphocyte–platelet (HALP) score = hemoglobin [g/L] × albumin [g/L] × lymphocyte count [/L]/platelet count [/L]; albumin–bilirubin index (ALBI) = (0.66 × log_10_[total bilirubin (μmol/L)]) + (−0.085 × albumin [g/L]) [14]; platelet–albumin–bilirubin index (PALBI) = (2.02 × log_10_ bilirubin) − (0.37 × [log_10_ bilirubin]^2^) − (0.04 × albumin) − (3.48 × log_10_ platelets) + (1.01 × [log_10_ platelets]^2^) [15]; platelet-to-lymphocyte ratio (PLR) = platelet count/lymphocyte count; and modified Glasgow Prognostic Score (mGPS) [16], scored as 0 for C-reactive protein (CRP) ≤ 10 mg/L, 1 for CRP > 10 mg/L with albumin ≥ 35 g/L, and 2 for CRP > 10 mg/L with albumin < 35 g/L. When laboratory units were reported in non-SI format, we applied the following standard conversions: bilirubin (mg/dL) × 17.1 = μmol/L; albumin (g/dL) × 10 = g/L.

The baseline CRP was measured within the same seven-day window applied to the other laboratory values and was available in 116 of 130 patients and in 109 of those with an assigned Mandard TRG. The mGPS distribution was 0 in 93 patients, 1 in 18, and 2 in 5. The mGPS 2 category was therefore sparse, and estimates involving it should be interpreted with caution.

Clinical T and N stage were not recorded in either institutional database and could not be reconstructed retrospectively with acceptable reliability; baseline disease burden was therefore not available for adjustment.

### 2.3. Outcomes

The primary endpoint was the association between pretreatment PNI values and major pathological response, defined as Mandard TRG 1–2. Disease-free survival (DFS) was the time from the first cycle of systemic therapy to the first documented locoregional recurrence, distant metastasis, or death from any cause—whichever occurred first. Overall survival (OS) was the time from diagnosis to death from any cause. For the secondary postoperative landmark analyses, we set the date of surgery as the time origin. We measured postoperative DFS from the date of surgery to the first occurrence of recurrence, distant metastasis, death, or the last disease-free follow-up. We measured postoperative OS from the date of surgery to death or the last clinical follow-up. The pathology report did not state a Mandard tumor regression grade for seven patients, who were excluded from pathological response analyses; survival analyses used the full cohort of 130 patients.

We defined major pathological response as Mandard tumor regression grade 1 or 2: complete regression with no residual tumor cells (TRG 1) or rare residual tumor cells scattered through fibrosis (TRG 2). This dichotomy follows the original description by Mandard et al. [17], which has been widely adopted in gastro-esophageal series because grades 1 and 2 together identify tumors in which fibrosis predominates over residual carcinoma. Grades 3, 4, and 5 denote, respectively, an increased number of residual cells with fibrosis still predominant, residual cancer outgrowing fibrosis, and absence of regressive change. We note that TRG 2 is not synonymous with pathological complete response, which we report separately as ypT0N0.

The pathological response analysis estimates the association between baseline biomarkers and pathological regression among patients who proceeded to curative-intent resection; it does not estimate response prediction among all patients starting perioperative therapy.

We measured disease-free survival from initiation of systemic therapy because the exposure of interest is a pretreatment biomarker, so anchoring at surgery would condition on an event occurring after the exposure. We acknowledge that disease-free survival is conventionally measured from definitive local treatment, and we therefore label the surgery-anchored analyses as postoperative disease-free survival throughout. Because case identification required a resection record, no patient in the analyzed cohort experienced an event between the initiation of chemotherapy and surgery, reflecting the selection mechanism rather than a true absence of pre-surgical events.

### 2.4. Statistical Analysis

We performed analyses using IBM SPSS Statistics version 27.0 and R version 4.5.1. Continuous variables are presented as mean ± standard deviation or median (interquartile range), as appropriate, and categorical variables as *n* (%). Groups were compared using the independent-samples *t*-test or Mann–Whitney U test for continuous variables and the chi-square or Fisher’s exact test for categorical variables. We evaluated associations between the 11 pretreatment inflammatory and nutritional indices and the five Mandard tumor regression grade (TRG) categories using the Kruskal–Wallis test, followed by Bonferroni-adjusted pairwise comparisons when appropriate. Comparisons for major pathological response (MPR; TRG 1–2 vs. TRG 3–5) used the Mann–Whitney U test.

Because we included all consecutive eligible patients treated during the predefined period, we did not perform an a priori power calculation, and biomarker analyses were exploratory. We examined each index separately to limit overfitting and did not impute missing data; we used complete cases and reported analysis-specific denominators (Supplementary Table S7). We applied the Benjamini–Hochberg procedure separately to the eleven index-specific ROC analyses, adjusted logistic regression models, and adjusted Cox models for each survival endpoint. We treated other exploratory *p* values as nominal. Events per variable were 28 across three covariates in the multivariable logistic model and 48 across three covariates in the Cox models. We did not perform a post hoc power calculation, as it provides no information beyond the reported confidence intervals. The assumed causal structure, including which variables were treated as confounders and which were mediators, is shown in Supplementary Figure S2.

We used conventional ROC analysis to assess discrimination for pathologic response. AUC confidence intervals and *p* values were calculated using the nonparametric DeLong method, and exploratory cutoffs were determined using the Youden index. We carried forward the PNI as the principal exploratory index because it had the numerically highest AUC among the eleven indices, not because its discrimination was statistically significant. We further examined the PNI AUC and data-derived cutoff using 1000 nonparametric bootstrap resamples. We estimated time-dependent AUCs for DFS at 12 and 24 months using inverse probability of censoring weighting, while Harrell’s c-index summarized overall discrimination. We used cutoffs derived by maximizing the Youden index only to describe classification performance for the response endpoint from which they were derived. We did not apply them to survival analysis because a threshold optimized in the same dataset yields optimistically biased log-rank statistics. We therefore modeled all index–survival associations with the index on its continuous scale.

We estimated median follow-up using the reverse Kaplan–Meier method, measuring DFS from the initiation of neoadjuvant therapy and OS from diagnosis. We estimated survival distributions using the Kaplan–Meier method and compared them with the log-rank test. Baseline Cox analyses evaluated each index separately, both unadjusted and adjusted for age and sex. We first examined postoperative variables in univariable landmark analyses, using surgery as the time origin. We considered variables associated with postoperative DFS at *p* < 0.10 for the exploratory multivariable model and used the same covariate set for the postoperative OS model. We modeled treatment completion as a time-dependent covariate, with the results reported as hazard ratios with 95% confidence intervals. All tests were two-sided, and we considered *p* < 0.05 nominally significant. We evaluated associations with MPR using univariable logistic regression and prespecified the multivariable adjustment set on clinical grounds rather than selecting it by univariable screening. It comprised CA19-9 level and signet-ring cell component, the two baseline variables most consistently reported to relate to pathological response in gastric cancer. Given 28 events, the model was deliberately restricted to three covariates. Clinical T and N stage were not available for adjustment (Section 2.2).

Internal validation used 1000 nonparametric bootstrap resamples. To avoid understating optimism, each resample repeated the full modeling process, including ranking the eleven indices by AUC, selecting the index with the highest value, and deriving its Youden threshold, rather than validating only the final model. We calculated optimism as the difference between the apparent performance in each resample and the performance of the resample-derived model applied to the original data. We assessed multivariable model calibration using the bootstrap calibration slope.

### 2.5. Ethics Statement

This study was conducted in accordance with the Declaration of Helsinki. The institutional review boards of all participating centers reviewed and approved the research protocol, and the Izmir City Hospital Ethics Committee granted primary ethical approval (Decision number: 2025/159; Date: 16 April 2025). We waived patient consent because the study was retrospective.

## 3. Results

### 3.1. Baseline Characteristics

Of the 151 records reviewed, 130 patients with gastric adenocarcinoma met the eligibility criteria (Figure 1). Their median age was 63 years (range 28–83), and 90 patients (69.2%) were male. The primary tumor was located in the proximal stomach (GEJ and cardia) in 45 patients (34.6%), the corpus in 57 (43.8%), and the distal stomach (antrum) in 28 (21.5%). Classified by histological subtype, mixed or not otherwise specified (NOS) carcinomas were the most common carcinomas (*n* = 53, 40.8%), followed by diffuse (*n* = 36, 27.7%), intestinal (*n* = 33, 25.4%), and mucinous (*n* = 8, 6.2%). Most patients received FLOT as the neoadjuvant backbone (*n* = 126, 96.9%), with four patients (3.1%) receiving FOLFOX. A Mandard TRG was assigned to 123 patients, while the pathology report did not state the grade for seven patients. Table 1 presents the full baseline characteristics, stratified by TRG group. No significant differences in clinicopathological or treatment characteristics were found between the TRG groups at baseline (*p* > 0.05).

### 3.2. Pathologic Response

TRG was strongly associated with post-treatment pathological stage: both ypT (*p* < 0.001) and ypN distributions (*p* = 0.002) differed significantly between the TRG 1–2 and TRG 3–5 groups. Pathological complete response (ypT0N0) occurred in six patients, all within the TRG 1–2 group (4.6% of the analyzed cohort; *p* < 0.001). Creatinine differed nominally between response groups (*p* = 0.024), and the albumin–globulin ratio was borderline (*p* = 0.053); neither was corrected for multiple comparisons, and both likely reflect chance.

Among the eleven pretreatment inflammatory and nutritional indices, the PNI showed a borderline significant difference between the response groups, with higher values in patients achieving TRG 1–2 (median 53.5 vs. 50.2; *p* = 0.054). Among the 119 patients with both an available PNI and an assigned Mandard TRG, including 30 with major pathological response (MPR), PNI showed weak discrimination for MPR (AUC 0.618, 95% CI 0.490–0.746; *p* = 0.070). Nevertheless, the PNI had the numerically highest AUC among the eleven indices and was therefore carried forward as the principal exploratory candidate for cutoff-based analyses. We based this selection on its relative numerical performance for MPR rather than on statistically significant discrimination or an association with survival. The comparative ROC results for all eleven indices are presented in Supplementary Table S1.

The Youden index identified an exploratory PNI cutoff of ≥53.15, reported as approximately 53 for practical purposes, yielding 53.3% sensitivity, 74.2% specificity, and an apparent Youden index of 0.275. Internal validation using 1000 bootstrap resamples produced a mean AUC of 0.618 (SD 0.065; BCa 95% CI 0.478–0.731) and a median cutoff of 53.80 (95% bootstrap range 50.00–56.95). After optimism correction, the Youden index decreased to 0.212. None of the eleven indices demonstrated statistically significant discrimination after correction for multiple testing (Supplementary Table S1).

Internal validation incorporating the full modeling process produced a mean optimism of 0.051 in the AUC of the selected index, giving an optimism-corrected AUC of 0.567. Across the 1000 resamples, the PNI was the index with the highest AUC in 493 (49.3%) resamples; the PLR was selected in 142 (14.2%) resamples, the SIRI was selected in 93 (9.3%) resamples, and each of the remaining indices in between nine and 66 resamples. The identity of the best-performing index is therefore unstable under resampling. For the three-variable multivariable model, the apparent AUC was 0.704 with a bootstrap optimism of 0.032, giving an optimism-corrected AUC of 0.672, and the bootstrap calibration slope was 0.825, consistent with overfitting.

In univariable logistic regression, PNI ≥ 53.125 was significantly associated with TRG 1–2 (OR 3.280, 95% CI 1.388–7.751; *p* = 0.007), and the PNI as a continuous variable was also significant (OR 1.085 per unit, 95% CI 1.003–1.174; *p* = 0.043). Age, sex, tumor location, histological subtype, grade, MMR status, FLOT exposure, dose reduction, and the other ten indices were not significant in the univariable analyses. CA19-9 ≥ 37 was not significantly associated with TRG status (OR 0.367, 95% CI 0.101–1.337; *p* = 0.128).

In separate index-specific models adjusted for CA19-9 level and signet-ring cell component, only the continuous PNI showed a nominal association with the MPR (adjusted OR 1.104 per unit increase, 95% CI 1.013–1.202; *p* = 0.024); LMR was of borderline significance (adjusted OR 1.342, 95% CI 0.966–1.863; *p* = 0.079). Neither association remained statistically significant after the Benjamini–Hochberg correction (q = 0.260 and q = 0.351, respectively) (Supplementary Table S2).

In the exploratory cutoff-based multivariable model containing PNI ≥ 53.125, CA19-9 ≥ 37 U/mL, and a signet-ring cell component (*n* = 112; 28 major pathological responses), PNI ≥ 53.125 remained nominally associated with major pathological response (adjusted OR 3.823, 95% CI 1.503–9.722; *p* = 0.005), whereas elevated CA19-9 and a signet-ring cell component did not (Table 2).

In a sensitivity analysis additionally adjusted for age, sex, tumor location, histological subtype, and signet-ring component, the direction and approximate magnitude of the PNI association were unchanged. However, the model is at the limit of what 28 events support, and the estimates are correspondingly imprecise.

We also analyzed tumor regression grade as an ordinal five-level variable. Across the eleven indices, the Kruskal–Wallis *p* values ranged from 0.033 for LMR to 0.885 for NMR, and no index remained significant after the Benjamini–Hochberg correction (all q ≥ 0.36). The Spearman correlation with the ordinal grade was nominally significant for the PNI (rho −0.202; *p* = 0.028), in the expected direction, but this also did not survive correction. The ordinal analysis therefore reproduces the dichotomized comparison and identifies no index missed by the binary analysis (Supplementary Table S5).

Formal comparison of correlated ROC curves showed that the PNI’s AUC did not differ significantly from that of any other index (all *p* ≥ 0.18; Supplementary Table S6). In the 104 patients with all eleven indices available, the PLR had a marginally higher point estimate than the PNI (0.617 vs. 0.613). The indices were also substantially interrelated, with seven pairs showing |Spearman rho| ≥ 0.70 (Supplementary Figure S3), reflecting their shared leukocyte, platelet, and albumin components.

### 3.3. Disease-Free Survival

During a median follow-up of 20.5 months from the initiation of systemic therapy (reverse Kaplan–Meier), 48 events were observed. The median follow-up measured from diagnosis for overall survival was 22.1 months, while median DFS was 29.0 months (95% CI, 20.7–50.5). In the Kaplan–Meier analysis, patients with TRG 1–2 had significantly longer DFS than those with TRG 3–5 (median, not reached vs. 20.8 months; log-rank *p* = 0.014; Supplementary Figure S1).

The pretreatment inflammatory and nutritional biomarkers discriminated poorly for DFS. At 12 months, time-dependent AUCs ranged from 0.464 to 0.612, and all 95% confidence intervals included 0.50. At 24 months, they ranged from 0.347 to 0.692; the confidence intervals excluded 0.50 for the SIRI (0.692, 95% CI 0.556–0.804) and the mGPS (0.600, 95% CI 0.506–0.696) and were in the opposite direction for the PALBI (0.347, 95% CI 0.191–0.480). Harrell’s c-indices ranged from 0.463 to 0.595. No index demonstrated consistent discrimination across the two time points (Supplementary Table S3).

Using the initiation of neoadjuvant therapy as the time origin, higher pretreatment PNI was associated with better DFS when modeled continuously (HR per 1-unit increase, 0.948; 95% CI, 0.903–0.994; *p* = 0.028). However, this association did not remain statistically significant in the baseline-only multivariable model including the PNI, age, and sex (*n* = 126; 47 events; adjusted HR per 1-unit increase, 0.954; 95% CI, 0.906–1.005; *p* = 0.075). Age and sex were not significantly associated with DFS. While the NLR and SII were nominally associated with DFS in univariable Cox analysis (NLR, HR 1.139 per unit, 95% CI 1.030–1.259, *p* = 0.011; SII, HR 1.00023, 95% CI 1.00001–1.00045, *p* = 0.042), neither association survived the Benjamini–Hochberg correction (q = 0.124 and q = 0.153, respectively). We carried the PNI forward to the main multivariable survival model as the principal exploratory candidate based on its numerically highest AUC for major pathological response. We evaluated all eleven indices in separate univariable and age- and sex-adjusted Cox models and report the complete results in Supplementary Table S4.

We examined post-treatment variables separately in a landmark analysis, using the surgery date as the time origin. In univariable analyses, TRG 1–2 was associated with better postoperative DFS than TRG 3–5 (HR, 0.359; 95% CI, 0.151–0.855; *p* = 0.021). ypN stage was strongly prognostic overall (*p* < 0.001): compared with ypN0, the hazard was higher for ypN2 (HR, 2.661; 95% CI, 0.985–7.191; *p* = 0.054) and ypN3 (HR, 5.052; 95% CI, 2.276–11.215; *p* < 0.001). ypT stage was significant overall (*p* < 0.001), though the category-specific estimates were imprecise.

In the postoperative multivariable model including TRG, categorical ypN stage, and time-dependent treatment completion (*n* = 122; 43 events), only ypN stage remained independently prognostic (overall *p* = 0.003). The adjusted HRs relative to ypN0 were 1.640 for ypN1 (95% CI, 0.625–4.297; *p* = 0.315), 2.268 for ypN2 (95% CI, 0.831–6.191; *p* = 0.110), and 4.421 for ypN3 (95% CI, 1.953–10.008; *p* < 0.001). Neither TRG 1–2 (adjusted HR, 0.525; 95% CI, 0.206–1.338; *p* = 0.177) nor treatment completion (adjusted HR, 0.688; 95% CI, 0.332–1.427; *p* = 0.315) remained independently associated with postoperative DFS (Table 3).

### 3.4. Overall Survival

A total of 34 deaths (26.2%) occurred during a median follow-up of 22.1 months, with a median OS of 38.8 months (95% CI 20.6–NE). In the Kaplan–Meier analysis, OS did not differ significantly between TRG subgroups: 33.2 months (95% CI 32.6–NE) for TRG 1–2 vs. 38.8 months (95% CI 27.5–NE) for TRG 3–5 (log-rank *p* = 0.334).

Using the date of diagnosis as the time origin, none of the evaluated baseline clinicopathological variables or the eleven pretreatment inflammatory and nutritional indices was significantly associated with OS in univariable Cox analyses. We carried forward the PNI as the principal exploratory candidate because it had the numerically highest AUC for major pathological response among the eleven indices. We evaluated all eleven indices in separate univariable and age- and sex-adjusted Cox models and report the complete results in Supplementary Table S4.

For the PNI, the univariable HR per 1-unit increase was 0.980 (95% CI, 0.923–1.041; *p* = 0.513). The PNI showed no association with OS in the baseline-only multivariable model including the PNI, age, and sex (*n* = 126; 33 deaths; adjusted HR per 1-unit increase, 0.997; 95% CI, 0.936–1.062; *p* = 0.930), and neither age (adjusted HR per 1-year increase, 1.035; 95% CI, 0.992–1.079; *p* = 0.116) nor female sex (adjusted HR, 0.877; 95% CI, 0.410–1.875; *p* = 0.735) was significantly associated with OS.

We again examined post-treatment variables separately from the date of surgery. In univariable analyses, ypT and ypN stages were significantly associated with postoperative OS overall (*p* = 0.008 and *p* = 0.011, respectively). Compared with ypN0, only ypN3 was associated with a significantly higher mortality hazard (HR, 4.071; 95% CI, 1.712–9.679; *p* = 0.001); ypN1 and ypN2 were not significant. Although ypT stage was significant overall, none of its individual categories differed significantly from ypT0, and the estimates were imprecise. Neither TRG 1–2 nor perioperative treatment completion modeled as a time-dependent covariate was significantly associated with postoperative OS.

In the postoperative multivariable model including TRG, categorical ypN stage, and time-dependent perioperative treatment completion (*n* = 123; 30 deaths), ypN stage remained independently prognostic overall (*p* = 0.016). The adjusted HRs relative to ypN0 were 1.368 for ypN1 (95% CI, 0.451–4.147; *p* = 0.580), 1.156 for ypN2 (95% CI, 0.292–4.577; *p* = 0.836), and 4.027 for ypN3 (95% CI, 1.564–10.367; *p* = 0.004). Neither TRG 1–2 (adjusted HR, 0.816; 95% CI, 0.305–2.182; *p* = 0.685) nor treatment completion (adjusted HR, 0.894; 95% CI, 0.346–2.311; *p* = 0.817) was independently associated with postoperative OS (Table 4).

Overall survival was 92.8% (95% CI 88.3–97.3) at 12 months, 70.2% (60.2–80.2) at 24 months, and 54.9% (40.3–69.4) at 36 months, with 108, 38, and 12 patients at risk, respectively. Disease-free survival was 81.1% (74.1–88.1), 51.6% (39.9–63.3), and 35.2% (19.4–51.0) at the same time points. The adjusted PNI estimate for overall survival, HR 0.997 per unit (95% CI 0.936–1.062), corresponds to HR 0.983 per standard deviation (95% CI 0.686–1.409); effects as large as a 31% reduction or a 41% increase in hazard per standard deviation therefore remain compatible with these data.

## 4. Discussion

In this study, we compared eleven baseline inflammatory and nutritional indices head to head against a uniformly graded pathological response endpoint and against survival in a real-world cohort of patients receiving perioperative chemotherapy and curative-intent surgery for gastric cancer [18,19,20]. The cohort’s median OS of 38.8 months and DFS of 29.0 months are consistent with contemporary real-world FLOT series, which typically report a median OS between 35 and 42 months. The modest difference relative to the FLOT4-AIO trial (median OS 50 months) likely reflects older patient demographics, a higher comorbidity burden, and the unselected nature of routine clinical practice [21]. The importance of real-world attrition is further highlighted by the finding that completion of the full perioperative regimen was associated with disease-free survival in the univariable analysis but was not independently associated with either endpoint once modeled as a time-dependent covariate, consistent with international observational data [22].

The observed pathological response distribution was broadly comparable with contemporary FLOT-treated cohorts, although direct numerical comparison requires caution. Six patients (4.6% of the analyzed cohort, 4.9% of those with an assigned grade) achieved a pathological complete response defined as ypT0N0, and 13 patients (10.6%) were graded Mandard TRG 1. These figures are not directly comparable with the 16% pathological complete regression rate reported in FLOT4-AIO [4], which used centrally assessed Becker criteria rather than the Mandard system; the two grading systems are not interchangeable, and their thresholds do not identify the same biological category. We note also that this comparison is descriptive benchmarking rather than evidence of treatment efficacy: the study has no untreated or alternative-treatment comparator, was not designed to estimate a treatment effect, and cannot exclude differences in patient selection between cohorts. Achieving TRG 1–2 was significantly associated with lower recurrence (12.5% vs. 34.1%; *p* = 0.023) and a longer DFS in the Kaplan–Meier analysis (log-rank *p* = 0.014), supporting TRG as a clinically meaningful surrogate endpoint of treatment efficacy [23].

Despite a superior DFS, TRG 1–2 patients had a numerically shorter median OS (33.2 vs. 38.8 months; *p* = 0.334) than the TRG 3–5 group. Immature follow-up at 22.1 months, the small size of the TRG 1–2 subgroup (*n* = 32), and the wide, right-censored OS confidence interval in this group (95% CI 32.6–NE) are plausible explanations, but the available data cannot distinguish between them, and we do not assert that the pattern will reverse with longer follow-up.

Perioperative practice at the two centers followed a selective rather than universal D2 policy over the study period, reflecting surgeon and center practice, tumor location (with proximal and gastro-esophageal junction tumors often managed with a modified nodal dissection), patient age and comorbidity, and the evolution of practice across the study period. The median number of nodes harvested was 30. We acknowledge that a D2 rate of 61.2% is lower than in contemporary European series and that this may affect nodal staging accuracy, which is relevant because ypN stage emerged as the strongest independent prognostic variable. Understaging in patients undergoing a less extensive dissection would be expected to bias the ypN–survival association toward the null, but stage migration cannot be excluded, and the magnitude of the ypN effect we report should therefore be interpreted with caution.

Our principal result is negative: in the ROC analysis, none of the eleven indices, including the PNI, showed statistically significant discrimination for pathological response, and internal validation incorporating index selection and threshold derivation gave an optimism-corrected area under the curve of 0.567 for the selected index. Against that background, the PNI was the only index nominally associated with major pathological response (TRG 1–2) in multivariable analysis and carried the highest point estimate, but its area under the curve of 0.618 (95% CI 0.490–0.746; *p* = 0.070) describes a modest signal rather than a discriminating test, and its advantage over the other indices was neither large nor statistically distinguishable. In univariable Cox modeling for DFS, higher pretreatment PNI was associated with a lower hazard (HR 0.948 per unit; *p* = 0.028), but this association was not confirmed in the baseline-adjusted model, and the PNI was not associated with OS under any specification. We therefore make no claim that the PNI predicts survival.

Nonetheless, we resist inferring that the PNI is the best predictor of outcome in advanced gastric cancer. NLR carries the largest meta-analytic evidence base for survival in this disease [24], while the PNI performs best in perioperative and surgical series [25], and the modified Glasgow Prognostic Score remains the most extensively validated inflammation-based score across solid tumors [16]. We therefore included both the PLR and mGPS in our panel so that the comparison would not be confined to the newer composite indices. Head-to-head comparisons within single cohorts are scarce, and cutoffs are not standardized [26]. Our contribution is the comparison itself, and its result is sobering: against a single, uniformly graded endpoint, no index discriminated adequately, and the PNI’s advantage over the others was small and not statistically significant. Neither the PLR nor the mGPS discriminated better than the ratio-based indices, and both showed the same pattern of weak, non-significant association seen across the panel.

The most substantial European data on the PNI in gastric cancer come from the GASTRODATA registry, in which the PNI was evaluated in 721 patients undergoing gastrectomy with multimodal treatment. A low PNI was strongly associated with 90-day mortality and adverse postoperative outcomes, and the optimal threshold identified was 45.5. Two differences from our own analysis are instructive. First, that study measured the PNI immediately before surgery, whereas we measured it before the start of systemic therapy, so the two values describe different clinical states: one after several months of chemotherapy and its nutritional consequences, and the other at presentation. Second, the threshold differs substantially from the ~53 value derived here. Together with the instability of our own threshold under resampling, this reinforces our position that data-derived PNI cutoffs are not transportable between cohorts. The GASTRODATA outcomes were postoperative and short-term, whereas our endpoint was pathological regression; evidence that the PNI relates to perioperative risk is considerably stronger than evidence that it predicts response to chemotherapy [27].

The eleven indices should not be regarded as eleven independent biological hypotheses; rather, they recombine a small number of underlying blood components, so our multiplicity correction is conservative.

The PNI combines a nutritional and an adaptive-immune term, which offers a conceptual reason why it might behave differently than the purely inflammatory ratios. We present this as an interpretation rather than a demonstrated mechanism: serum albumin is influenced by inflammation, hepatic synthesis, and hydration, as well as by nutrition, and the peripheral lymphocyte count does not measure intratumoral adaptive immunity [8,28,29,30,31].

One possible explanation for the failure of peripheral indices is that pathological regression is a tissue-level immunological event. Tertiary lymphoid structures, which function as sites of local antigen presentation and T-cell priming [32], are associated with improved prognosis and with response to checkpoint blockade, and in gastric cancer their density correlates with CD8 infiltration and survival [33,34]. We did not measure tertiary lymphoid structures, CD8, or cytokines, so this remains an untested hypothesis; archived tissue is available for the whole cohort, making it directly testable in future work.

Turning to clinical implications, the PNI has the practical advantage of requiring just two universally available and inexpensive measurements—serum albumin and peripheral lymphocyte count—so it could, in principle, be computed in any clinical setting worldwide. We nonetheless make it clear that our data do not support using it to select treatment. Discrimination was modest and did not reach statistical significance; we derived and validated the threshold of approximately 53 internally in the same cohort in which we tested it; and the cohort is retrospective, single-country, and restricted to patients who underwent surgery. A pretreatment PNI at or above approximately 53 may identify patients who are nutritionally and immunologically better placed to achieve pathological downstaging, but this is a hypothesis for prospective testing, not a criterion for clinical decision-making, and no threshold should be applied to individual patients before external validation in geographically and clinically distinct cohorts.

These findings are relevant to the current era of perioperative immunotherapy. While the MATTERHORN trial has established the benefit of adding durvalumab to FLOT [5], the cost-effectiveness of universal immunotherapy adoption remains uncertain [6]. Our cohort was treated before perioperative immunotherapy entered routine practice at our centers, so these data cannot address whether the PNI predicts benefit from checkpoint blockade, and we make no such claim.

We examined whether baseline indices predicted delivery of the planned perioperative regimen in our own cohort and found that they did not. Pretreatment PNI was not associated with treatment completion (odds ratio 1.067 per unit, 95% CI 0.984–1.157; *p* = 0.119), and the median PNI did not differ between patients who completed therapy and those who did not (51.0 vs. 49.2; *p* = 0.277); NLR, SII, HALP, and serum albumin were likewise unassociated. This negative finding constrains interpretation in a useful way: it provides no support for the intuitive hypothesis that baseline nutritional status governs treatment delivery, and it argues against treatment completion mediating the effect of the PNI on disease-free survival. We did not measure body composition, which is a limitation; however, all patients underwent preoperative CT staging, so the skeletal muscle index is retrievable from existing imaging, and we regard that analysis as a natural extension of this work.

Perioperative treatment completion was associated with DFS in the univariable analysis but was not independently associated with either endpoint once modeled as a time-dependent covariate, so we do not present it as a prognostic factor. The observation that most patients who complete therapy do better nevertheless underlines the value of proactive nutritional support, toxicity management, and prehabilitation, even though our data cannot establish that completion itself confers benefit. Because the baseline PNI reflects both nutritional reserve and immune competence, it may be a convenient summary measure of a modifiable state. We emphasize, however, that appropriate intervention targets are malnutrition, systemic inflammation, and functional status, not the index itself. The prospective evaluation of nutritional and prehabilitation interventions in this setting is warranted, with the PNI or a similar composite used as an exploratory intermediate biomarker of response to those interventions rather than as the object of treatment.

### Limitations

This study has several limitations. The retrospective design introduces inherent risks of selection bias and residual confounding that multivariable adjustment cannot fully address. Because eligibility requires completed curative-intent resection, the cohort represents a surgery-completer population. Of 132 patients documented to have received at least one cycle of neoadjuvant chemotherapy, only one did not proceed to resection. This is far lower than the attrition reported in prospective neoadjuvant series and reflects a case identification strategy rather than an unusually favorable treatment course: patients who began treatment and never reached surgery are systematically underrepresented in surgical and oncology databases, and at least three of the ten patients with unretrievable records are documented to have received neoadjuvant chemotherapy. The sample size of 130 patients limits statistical power, particularly for subgroup analyses and modest OS associations, with only 34 deaths recorded at current follow-up and 48 disease-free survival events observed. The use of two neoadjuvant regimens, predominantly FLOT with a small proportion of FOLFOX, introduces therapeutic heterogeneity, though their distribution was balanced across TRG groups. Immature follow-up and differential censoring plausibly explain the numerically shorter overall survival in the TRG 1–2 group, but the available data cannot distinguish between them, and we do not assert that the pattern will reverse with longer follow-up. The study was conducted in a middle-income healthcare setting, and differences in perioperative support and toxicity management may limit generalizability to higher-resourced systems. Tumor regression grading was performed locally at each institution without central pathological review, introducing potential interobserver variability in TRG classification. Seven patients lacked a Mandard grade in the pathology report and were excluded from pathological response analyses, while the baseline CRP was unavailable in 14 patients, limiting the mGPS analysis. We assessed inflammatory and nutritional indices at baseline only and did not systematically perform longitudinal post-neoadjuvant profiling, which could better capture treatment-induced immunomodulation. Finally, we derived the PNI cutoff values from the same cohort in which we evaluated them. To mitigate the resulting optimism bias, we performed internal validation using a 1000-resample bootstrap with bias-corrected and accelerated (BCa) confidence intervals; optimism was modest for the Youden index (0.063), but the fuller internal validation, which repeated index selection and threshold derivation within each resample, gave an optimism-corrected area under the curve of 0.567 against an apparent 0.618. Nonetheless, internal validation cannot substitute for prospective external validation in geographically and clinically distinct cohorts, which remains necessary before clinical implementation. The retrospective design also limited our ability to collect anthropometric data, such as BMI and ASA functional status scores.

The most important limitation of the adjusted analyses is the lack of baseline clinical stage data: neither institutional database captured clinical T and N stage, so the models could not adjust for pretreatment disease burden. This is a material omission because tumor stage plausibly influences both systemic nutritional and inflammatory status and the probability of pathological regression and could therefore confound the association we report between the PNI and major pathological response. Accordingly, the associations described here should be read as unadjusted for disease burden, and any confirmatory study should record cT and cN prospectively.

Only 61.2% of patients underwent D2 lymphadenectomy, and the number of nodes examined varied. Nodal understaging in patients with less extensive dissection may have caused stage migration, limiting the precision of ypN estimates.

Because the cohort was identified through surgical records, the analysis is conditional on having reached resection. If baseline nutritional or inflammatory status influences the probability of reaching surgery, this conditioning can induce collider bias in the relationship between baseline indices and pathological response, while also limiting generalizability. The estimand is therefore the association between baseline biomarkers and pathological regression among patients who underwent curative-intent resection.

Mandard grading was performed locally at each institution without central review or a study-specific protocol, and no interobserver agreement statistic is available. With only 32 major responses, even modest misclassification could materially affect the estimated discrimination and odds ratio. We did not assign a Mandard grade in seven patients, all of whom were treated at one of the two centers (9.5% vs. 0%; *p* = 0.019), suggesting that missingness reflects differing reporting practices rather than a random process. The distribution across the five grades did not differ significantly between centers (*p* = 0.084), and the major pathological response rate was 21 of 67 vs. 11 of 56 (*p* = 0.155); these comparisons do not exclude interobserver variation but provide no evidence of gross systematic difference.

Missing data were not randomly distributed: the baseline CRP, which determines the mGPS, was unavailable in 2.7% of patients at one center and 21.4% at the other (*p* = 0.001), and all seven patients without an assigned Mandard grade were treated at a single center (*p* = 0.019). Patients with and without a CRP measurement had a similar median PNI (50.8 vs. 50.4; *p* = 0.939) and CA19-9 (11.5 vs. 12.8; *p* = 0.782), but the major pathological response rate differed (28.4% vs. 7.1%), indicating that CRP missingness is not independent of the outcome. The mGPS analyses in particular are therefore conducted in a subgroup that is not representative of the whole cohort. Differing denominators between indices also complicate direct comparison, since apparent differences in performance may partly reflect analysis in different subsets.

## 5. Conclusions

In this two-center retrospective cohort of 130 patients receiving perioperative chemotherapy for locally advanced gastric or gastro-esophageal junction adenocarcinoma, eleven baseline inflammatory and nutritional indices were compared head-to-head against a uniformly graded pathological response endpoint and against survival. Although the PNI was the only index nominally associated with major pathological response, its discrimination was modest and not statistically significant, and no index in the panel was associated with overall survival. No association survived correction for multiple comparisons. These indices, as a group, are not accurate enough to guide treatment decisions in this setting, and the PNI threshold identified here should be regarded as a hypothesis for prospective evaluation rather than a criterion for clinical use. External validation in geographically and clinically distinct cohorts is required before applying any blood-based index to individual patients.

## Figures and Tables

**Table 1:** Baseline clinicopathological and treatment characteristics stratified by Mandard tumor regression grade.

Variable	All Patients (*n* = 130)	TRG 1–2 (*n* = 32)	TRG 3–5 (*n* = 91)	*p* Value
Age (range)	63 (28–83)	61.5 (28–79)	63.0 (31–83)	0.550
Sex				0.783
Male	90 (69.2%)	24 (75.0%)	64 (70.3%)	
Female	40 (30.8%)	8 (25.0%)	27 (29.7%)	
Tumor localization				0.679
Proximal (GEJ + Cardia)	45 (34.6%)	14 (43.8%)	30 (33.0%)	
Corpus	57 (43.8%)	13 (40.6%)	40 (44.0%)	
Distal (Antrum)	28 (21.5%)	5 (15.6%)	21 (23.1%)	
Histological subtype				0.407
Intestinal	33 (25.4%)	11 (34.4%)	20 (22.0%)	
Diffuse	36 (27.7%)	6 (18.8%)	29 (31.9%)	
Mucinous	8 (6.2%)	2 (6.2%)	5 (5.5%)	
Mixed/NOS	53 (40.8%)	13 (40.6%)	37 (40.7%)	
Signet-ring cell component	28 (21.5%)	4 (12.5%)	23 (25.3%)	0.210
Tumor grade (G1–2 vs. G3) †				0.198
G1–2	38/83 (45.8%)	12/20 (60.0%)	23/58 (39.7%)	
G3	45/83 (54.2%)	8/20 (40.0%)	35/58 (60.3%)	
MMR status †				1.000
Proficient (pMMR)	85/93 (91.4%)	20/22 (90.9%)	60/66 (90.9%)	
Loss of any protein (dMMR)	8/93 (8.6%)	2/22 (9.1%)	6/66 (9.1%)	
Neoadjuvant regimen				0.572
FLOT	126 (96.9%)	32 (100%)	87 (95.6%)	
FOLFOX	4 (3.1%)	0 (0.0%)	4 (4.4%)	
Number of median neoadjuvant cycles (range)	4 (1–8)	4 (2–8)	4 (1–8)	0.747
Perioperative treatment completed	108 (83.1%)	28 (87.5%)	74 (81.3%)	0.599
Dose reduction	29 (22.3%)	10 (31.2%)	17 (18.7%)	0.219
D2 lymphadenectomy ‡	79 (61.2%)	20 (62.5%)	55 (60.4%)	1.000
ypT stage				<0.001
ypT0	7 (5.4%)	7 (21.9%)	0 (0.0%)	
ypT1	14 (10.8%)	6 (18.8%)	7 (7.7%)	
ypT2	14 (10.8%)	5 (15.6%)	8 (8.8%)	
ypT3	56 (43.1%)	13 (40.6%)	39 (42.9%)	
ypT4	39 (30.0%)	1 (3.1%)	37 (40.7%)	
ypN stage				0.002
ypN0	51 (39.2%)	18 (56.3%)	28 (30.8%)	
ypN1	29 (22.3%)	10 (31.3%)	17 (18.7%)	
ypN2	19 (14.6%)	2 (6.3%)	17 (18.7%)	
ypN3	31 (23.8%)	2 (6.3%)	29 (31.9%)	
Pathological complete response (ypT0N0)	6 (4.6%)	6 (18.8%)	0 (0.0%)	<0.001
Recurrence	37 (28.5%)	4 (12.5%)	31 (34.1%)	0.023
Pretreatment PNI, median (range)	50.47 (31.40–64.30)	53.47 (41.90–62.40)	50.20 (31.40–64.30)	0.054
Pretreatment NLR, median (range)	2.57 (0.91–13.17)	2.50 (0.91–8.90)	2.60 (1.12–13.17)	0.394
Pretreatment SII, median (range)	702.57 (179.72–7772.12)	635.60 (179.72–2914.15)	730.05 (263.64–7772.12)	0.306
Pretreatment PLR, median (range)	148.33 (65.22–1134.62)	125.97 (65.80–380.00)	151.84 (65.22–1134.62)	0.176
Pretreatment mGPS §				0.398
0	93 (80.2%)	27 (87.1%)	62 (79.5%)	
1	18 (15.5%)	4 (12.9%)	12 (15.4%)	
2	5 (4.3%)	0 (0.0%)	4 (5.1%)	

Data are *n* (%) unless otherwise stated. A Mandard TRG was not stated in the pathology report for seven patients, who appear only in the all-patients column. † Valid cases only (denominators shown). ‡ One patient with missing D2 dissection status. § mGPS calculable in 116 patients overall and in 109 with an assigned TRG. *p*-values: Mann–Whitney U for continuous variables, chi-square or Fisher’s exact test for categorical variables. NOS, not otherwise specified; GEJ, gastro-esophageal junction; PNI, Prognostic Nutritional Index; NLR, neutrophil-to-lymphocyte ratio; SII, systemic immune–inflammation index; TRG, tumor regression grade.

**Table 2:** Multivariable logistic regression model associated with major pathological response (TRG 1–2).

Variable	*n* (Events)	Adjusted OR	95% CI	*p*-Value
PNI ≥ 53.125 (high vs. low)	112 (28)	3.823	1.503–9.722	0.005
CA19-9 ≥ 37 (high vs. low)	112 (28)	0.518	0.135–1.994	0.339
Signet-ring cell component	112 (28)	0.392	0.113–1.360	0.140

PNI: Prognostic Nutritional Index.

**Table 3:** Multivariable Cox regression analysis for disease-free survival.

Variable	*n* (Events)	HR	95% CI	*p*-Value
Panel A. Baseline prognostic analysis, from initiation of systemic therapy, pretreatment predictors only.				
PNI, per 1-unit increase	126 (47)	0.954	0.906–1.005	0.075
Female vs. male	126 (47)	1.038	0.554–1.945	0.908
Age, per 1-year increase	126 (47)	1.013	0.978–1.048	0.476
Panel B. Postoperative landmark analysis, from surgery, conditional on resection.				
Perioperative treatment completed (yes vs. no), time dependent *	122 (43)	0.688	0.332–1.427	0.315
Panel C. Post-treatment prognostic analysis using TRG and ypTNM.				
TRG 1–2 vs. TRG 3–5	122 (43)	0.525	0.206–1.338	0.177
ypN stage, overall				0.003
ypN1 vs. ypN0	122 (43)	1.640	0.625–4.297	0.315
ypN2 vs. ypN0	122 (43)	2.268	0.831–6.191	0.110
ypN3 vs. ypN0	122 (43)	4.421	1.953–10.008	<0.001

* Perioperative treatment completion was modeled as a time-dependent covariate. HR: hazard ratio; CI: confidence interval. PNI: Prognostic Nutritional Index; TRG: tumor regression grade.

**Table 4:** Multivariable Cox regression analysis for overall survival.

Variable	*n* (Events)	Adjusted HR	95% CI	*p*-Value
Panel A. Baseline prognostic analysis, from initiation of systemic therapy, pretreatment predictors only.				
PNI, per 1-unit increase	126 (33)	0.997	0.936–1.062	0.930
Female vs. male	126 (33)	0.877	0.410–1.875	0.735
Age, per 1-year increase	126 (33)	1.035	0.992–1.079	0.116
Panel B. Postoperative landmark analysis, from surgery, conditional on resection.				
Perioperative treatment completed, time-dependent *	123 (30)	0.894	0.346–2.311	0.817
Panel C. Post-treatment prognostic analysis using TRG and ypTNM.				
TRG 1–2 vs. TRG 3–5	123 (30)	0.816	0.305–2.182	0.685
ypN stage (overall)				0.016
ypN1 vs. ypN0	123 (30)	1.368	0.451–4.147	0.580
ypN2 vs. ypN0	123 (30)	1.156	0.292–4.577	0.836
ypN3 vs. ypN0	123 (30)	4.027	1.564–10.367	0.004

* Perioperative treatment completion was modeled as a time-dependent covariate. HR: hazard ratio; CI: confidence interval.

## Data Availability

The data from this study are available from the corresponding author upon reasonable request. The analysis code used to compute all eleven indices is provided as Supplementary Data S1.

## Supplementary Materials

The following supporting information can be downloaded at: https://www.mdpi.com/article/10.3390/jcm15176893/s1. Table S1: Receiver operating characteristic analyses of eleven pretreatment inflammatory and nutritional indices for major pathological response; Table S2: Univariable and multivariable logistic regression analyses of the eleven indices for major pathological response; Table S3: Time-dependent discrimination of the eleven indices for disease-free survival; Table S4: Univariable and age- and sex-adjusted Cox regression analyses of the eleven indices for disease-free and overall survival; Figure S1: Kaplan–Meier curves for disease-free survival by tumor regression grade group and by ypN stage; Table S5: Association between the eleven indices and Mandard tumor regression grade analyzed as an ordinal five-level outcome; Table S6: Pairwise comparison of correlated receiver operating characteristic curves, PNI versus each remaining index; Table S7: Missing baseline data by variable and by institution; Figure S2: Directed acyclic graph for the association between baseline PNI and major pathological response; Figure S3: Spearman correlation matrix of the eleven indices; Data S1: Analysis code used to compute all eleven indices.

## Abbreviations

The following abbreviations are used in this manuscript:

ALBI	Albumin–Bilirubin Index
AUC	Area Under Curve
BCa	Bias-Corrected and Accelerated
BH	Benjamini–Hochberg
CI	Confidence Interval
CRP	C-Reactive Protein
CT	Computed Tomography
DFS	Disease-Free Survival
FLOT	5-Fluorouracil, Leucovorin, Oxaliplatin, and Docetaxel
GEJ	Gastro-Esophageal Junction
HALP	Hemoglobin–Albumin–Lymphocyte–Platelet
HR	Hazard Ratio
IQR	Interquartile Range
LMR	Lymphocyte-to-Monocyte Ratio
mGPS	Modified Glasgow Prognostic Score
MPR	Major Pathological Response
NE	Not Estimable
NLR	Neutrophil-to-Lymphocyte Ratio
NMR	Neutrophil-to-Monocyte Ratio
NOS	Not Otherwise Specified
OR	Odds Ratio
OS	Overall Survival
PALBI	Platelet–Albumin–Bilirubin Index
pCR	Pathological Complete Response
PD-L1	Programmed Death Ligand-1
PLR	Platelet-to-Lymphocyte Ratio
PNI	Prognostic Nutritional Index
ROC	Receiver Operating Characteristic
SII	Systemic Immune–Inflammation Index
SIRI	Systemic Inflammatory Response Index
TRG	Tumor Regression Grade
