# Supplementary material for: Baseline Inflammatory and Nutritional Indices Show Limited Discrimination for Major Pathological Response and Survival After Neoadjuvant Chemotherapy for Gastric Cancer: A Retrospective Comparison of Eleven Indices
Source: J Clin Med. 2026 Sep 6;15(17):6893. doi: 10.3390/jcm15176893 (PMC13565757; doi:10.3390/jcm15176893)
Supplement: Supplementary file 1 [file jcm-15-06893-s001.zip › jcm-4510870-supplementary.pdf]

Journal of
Clinical Medicine

MDPI

Supplementary Materials

Baseline Inflammatory and Nutritional Indices Show Limited Discrimination for Major Pathological Response and Survival After Neoadjuvant Chemotherapy for Gastric Cancer: A Retrospective Comparison of Eleven Indices

Table S1. Receiver operating characteristic analyses of eleven pretreatment inflammatory and nutritional indices for major pathological response.

Index	N (MPR)	Direction	AUC (95% CI)	*p*	Cut-off	Sens.	Spec.	BH q
PNI	119 (30)	Higher	0.618 (0.490–0.746)	0.070	≥53.15	53.3%	74.2%	0.770
PLR	123 (32)	Lower	0.581 (0.461–0.701)	0.188	≤130.0	56.2%	69.2%	0.770
ALBI	115 (30)	Lower	0.566 (0.442–0.690)	0.298	≤−3.149	33.3%	82.4%	0.770
LMR	123 (32)	Higher	0.562 (0.444–0.679)	0.303	≥4.800	25.0%	91.2%	0.770
SIRI	123 (32)	Lower	0.562 (0.444–0.681)	0.301	≤0.700	18.8%	93.4%	0.770
SII	123 (32)	Lower	0.561 (0.441–0.681)	0.318	≤598.09	50.0%	68.1%	0.770
NLR	123 (32)	Lower	0.551 (0.429–0.673)	0.413	≤2.000	37.5%	76.9%	0.839
HALP	119 (30)	Higher	0.551 (0.417–0.684)	0.458	≥41.92	53.3%	68.5%	0.839
mGPS	109 (31)	Lower	0.541 (0.468–0.615)	0.271	≤0	87.1%	20.5%	0.770
PALBI	115 (30)	Lower	0.511 (0.393–0.628)	0.860	≤−2.144	96.7%	14.1%	0.953
NMR	123 (32)	Lower	0.503 (0.390–0.617)	0.953	≤10.500	87.5%	24.2%	0.953

MPR, major pathological response (Mandard TRG 1–2). Analyses were restricted to patients with an assigned Mandard tumor regression grade; denominators vary with laboratory data availability. The direction column indicates whether higher or lower index values were treated as indicating major pathological response. AUC confidence intervals and *p* values were calculated using the DeLong method against a null AUC of 0.50. Cut-offs were derived by maximizing the Youden index and are exploratory. The Benjamini–Hochberg (BH) procedure was applied to the eleven *p* values. Rows are ordered by AUC.

Table S2. Univariable and multivariable logistic regression analyses of the eleven indices for major pathological response.

Index	Crude N (MPR)	Crude OR (95% CI)	*p*	Adj. N (MPR)	Adjusted OR (95% CI)	*p*	BH q
PNI, per 1-unit	119 (30)	1.085 (1.003–1.174)	0.043	112 (28)	1.104 (1.013–1.202)	0.024	0.260
LMR, per 1-unit	123 (32)	1.289 (0.942–1.763)	0.113	116 (30)	1.342 (0.966–1.863)	0.079	0.351
HALP, per 1-unit	119 (30)	1.010 (0.991–1.030)	0.298	112 (28)	1.015 (0.995–1.037)	0.148	0.351
mGPS, per 1-point	109 (31)	0.537 (0.191–1.505)	0.237	104 (29)	0.425 (0.127–1.423)	0.165	0.351
ALBI, per 1-unit	115 (30)	0.504 (0.153–1.667)	0.262	108 (28)	0.440 (0.127–1.529)	0.197	0.351
PLR, per 1-unit	123 (32)	0.9973 (0.9923–1.0023)	0.293	116 (30)	0.9966 (0.9912–1.0020)	0.213	0.351
SIRI, per 1-unit	123 (32)	0.835 (0.614–1.135)	0.249	116 (30)	0.842 (0.623–1.139)	0.265	0.351
NLR, per 1-unit	123 (32)	0.888 (0.712–1.107)	0.290	116 (30)	0.886 (0.714–1.099)	0.272	0.351
SII, per 1-unit	123 (32)	0.9997 (0.9991–1.0003)	0.291	116 (30)	0.9997 (0.9991–1.0003)	0.287	0.351
PALBI, per 1-unit	115 (30)	0.999 (0.404–2.470)	0.998	108 (28)	0.893 (0.350–2.278)	0.812	0.894
NMR, per 1-unit	123 (32)	0.995 (0.895–1.107)	0.927	116 (30)	1.001 (0.903–1.109)	0.992	0.992

Each index was examined separately, both unadjusted (crude) and in a multivariable model adjusted for CA19-9 level (≥37 vs. <37 U/mL) and signet-ring cell component (present vs. absent).

Continuous indices were entered in their original units; mGPS was analyzed per one-point increase. The Benjamini–Hochberg (BH) procedure was applied to the eleven *p* values from the adjusted models. Rows are ordered by adjusted *p* value. OR, odds ratio.

Table S3. Time-dependent discrimination of the eleven indices for disease-free survival.

Index	N (Events)	Risk Direction	12-Month AUC (95% CI)	24-Month AUC (95% CI)	Harrell's c (95% CI)
PNI	126 (47)	Lower = higher risk	0.559 (0.426–0.682)	0.522 (0.369–0.686)	0.561 (0.467–0.645)
NLR	130 (48)	Higher = higher risk	0.557 (0.417–0.693)	0.605 (0.461–0.758)	0.562 (0.461–0.669)
NMR	130 (48)	Higher = higher risk	0.479 (0.341–0.631)	0.571 (0.416–0.736)	0.512 (0.408–0.618)
LMR	130 (48)	Lower = higher risk	0.612 (0.491–0.727)	0.630 (0.456–0.788)	0.595 (0.512–0.681)
PLR	130 (48)	Higher = higher risk	0.557 (0.413–0.692)	0.568 (0.414–0.715)	0.584 (0.477–0.686)
SII	130 (48)	Higher = higher risk	0.505 (0.369–0.655)	0.623 (0.464–0.759)	0.555 (0.459–0.672)
SIRI	130 (48)	Higher = higher risk	0.569 (0.429–0.699)	0.692 (0.556–0.804)	0.586 (0.491–0.683)
HALP	126 (47)	Lower = higher risk	0.491 (0.336–0.645)	0.470 (0.315–0.621)	0.523 (0.416–0.612)
ALBI	122 (46)	Higher = higher risk	0.512 (0.392–0.630)	0.390 (0.232–0.574)	0.506 (0.416–0.601)
PALBI	122 (46)	Higher = higher risk	0.464 (0.331–0.612)	0.347 (0.191–0.480)	0.463 (0.360–0.575)
mGPS	116 (40)	Higher = higher risk	0.510 (0.423–0.627)	0.600 (0.506–0.696)	0.528 (0.467–0.606)

Time-dependent cumulative/dynamic AUCs were estimated at 12 and 24 months using inverse probability of censoring weighting; Harrell's c-index summarizes overall discrimination. Confidence intervals are percentile intervals from 400 bootstrap resamples. Each index was oriented so that higher values indicate higher predicted risk; PNI, LMR and HALP were therefore reversed, as indicated in the risk direction column. Disease-free survival was measured from the initiation of neoadjuvant therapy.

Table S4. Univariable and age- and sex-adjusted Cox regression analyses of the eleven indices for disease-free and overall survival.

Panel A. Disease-free survival, measured from initiation of neoadjuvant therapy.

Index	N (events)	Crude HR (95% CI)	*p*	Adjusted HR (95% CI)	*p*	BH q
PNI, per 1-unit	126 (47)	0.948 (0.904–0.994)	0.028	0.954 (0.906–1.005)	0.075	0.276
NLR, per 1-unit	130 (48)	1.139 (1.030–1.259)	0.011	1.126 (1.015–1.248)	0.025	0.272
SII, per 1-unit	130 (48)	1.00023 (1.00001–1.00045)	0.042	1.00023 (0.99999–1.00047)	0.060	0.276
LMR, per 1-unit	130 (48)	0.873 (0.728–1.046)	0.141	0.889 (0.748–1.057)	0.183	0.384
PLR, per 1-unit	130 (48)	1.0014 (0.9996–1.0032)	0.122	1.0013 (0.9994–1.0032)	0.173	0.384
PALBI, per 1-unit	122 (46)	0.812 (0.454–1.452)	0.482	0.676 (0.361–1.267)	0.222	0.384
mGPS, per 1-point	116 (40)	1.471 (0.885–2.447)	0.137	1.370 (0.806–2.327)	0.245	0.384
ALBI, per 1-unit	122 (46)	1.343 (0.595–3.034)	0.478	1.089 (0.432–2.746)	0.856	0.942
HALP, per 1-unit	126 (47)	0.994 (0.978–1.010)	0.435	0.997 (0.981–1.014)	0.737	0.939
SIRI, per 1-unit	130 (48)	1.025 (0.940–1.117)	0.578	1.013 (0.928–1.107)	0.769	0.939
NMR, per 1-unit	130 (48)	1.003 (0.954–1.054)	0.914	1.001 (0.953–1.051)	0.974	0.974

Panel B. Overall survival, measured from diagnosis.

Index	N (events)	Crude HR (95% CI)	*p*	Adjusted HR (95% CI)	*p*	BH q
NLR, per 1-unit	130 (34)	1.124 (0.992–1.272)	0.067	1.103 (0.972–1.253)	0.130	0.759
HALP, per 1-unit	126 (33)	1.005 (0.987–1.023)	0.628	1.011 (0.992–1.030)	0.254	0.759

Index	N (events)	Crude HR (95% CI)	*p*	Adjusted HR (95% CI)	*p*	BH q
mGPS, per 1-point	116 (30)	1.450 (0.790–2.662)	0.231	1.337 (0.708–2.522)	0.371	0.759
PALBI, per 1-unit	122 (33)	0.881 (0.448–1.732)	0.714	0.722 (0.351–1.484)	0.375	0.759
SII, per 1-unit	130 (34)	1.00019 (0.99984–1.00054)	0.292	1.00015 (0.99982–1.00048)	0.378	0.759
LMR, per 1-unit	130 (34)	0.914 (0.756–1.106)	0.355	0.930 (0.782–1.107)	0.414	0.759
ALBI, per 1-unit	122 (33)	1.046 (0.401–2.726)	0.927	0.701 (0.229–2.144)	0.533	0.837
PLR, per 1-unit	130 (34)	1.0007 (0.9980–1.0034)	0.607	1.0004 (0.9974–1.0035)	0.793	0.930
SIRI, per 1-unit	130 (34)	1.026 (0.925–1.138)	0.633	1.011 (0.910–1.122)	0.841	0.930
NMR, per 1-unit	130 (34)	0.998 (0.938–1.062)	0.939	0.995 (0.937–1.056)	0.865	0.930
PNI, per 1-unit	126 (33)	0.980 (0.923–1.041)	0.513	0.997 (0.936–1.062)	0.930	0.930

Each index was examined separately, both unadjusted (crude) and adjusted for age and sex. All estimates are per one-unit increase in the index; mGPS was analyzed per one-point increase. Indices are entered on their original scales, so hazard ratios are not comparable in magnitude between indices measured on different scales. The Benjamini–Hochberg (BH) procedure was applied separately within each panel to the eleven *p* values from the adjusted models. Rows are ordered by adjusted *p* value. HR, hazard ratio.

Baseline Inflammatory and Nutritional Indices Show Limited Discrimination for Major Pathological Response and Survival after Neoadjuvant Chemotherapy for Gastric Cancer: A Retrospective Comparison of Eleven Indices

Table S5. Association between the eleven indices and Mandard tumor regression grade analyzed as an ordinal five-level outcome.

Index	n	Kruskal–Wallis *p*	Spearman rho	*p*	BH q
LMR	123	0.033	−0.173	0.056	0.364
PLR	123	0.173	+0.176	0.052	0.694
SIRI	123	0.252	+0.121	0.183	0.694
PNI	119	0.264	−0.202	0.028	0.694
PALBI	115	0.340	+0.051	0.586	0.694
NLR	123	0.387	+0.106	0.241	0.694
HALP	119	0.442	−0.115	0.211	0.694
mGPS	109	0.555	+0.085	0.377	0.699
ALBI	115	0.572	+0.120	0.202	0.699
SII	123	0.825	+0.096	0.293	0.885
NMR	123	0.885	−0.012	0.892	0.885

Analyses restricted to the 123 patients with an assigned Mandard grade; denominators vary with laboratory data availability. Grade distribution: TRG 1, n = 13; TRG 2, n = 19; TRG 3, n = 56; TRG 4, n = 25; TRG 5, n = 10. Kruskal–Wallis tests compare index distributions across the five grades. Spearman correlations treat grade as an ordinal variable, so a negative coefficient indicates that higher index values accompany lower (more favorable) grades. We applied the Benjamini–Hochberg (BH) procedure to the eleven Kruskal–Wallis *p* values. No index remained significant after correction. Rows are ordered by Kruskal–Wallis *p* value.

Table S6. Pairwise comparison of correlated receiver operating characteristic curves, PNI versus each remaining index.

Index	AUC	ΔAUC vs. PNI	SE (Δ)	z	*p*
PNI	0.613	—	—	—	—
PLR	0.617	−0.005	0.054	−0.09	0.932
SII	0.582	+0.031	0.060	0.52	0.602
LMR	0.566	+0.047	0.055	0.86	0.391
ALBI	0.566	+0.046	0.046	1.02	0.310
SIRI	0.564	+0.049	0.070	0.70	0.481
NLR	0.563	+0.050	0.056	0.89	0.372
HALP	0.561	+0.051	0.051	1.01	0.311
mGPS	0.541	+0.071	0.067	1.06	0.289
PALBI	0.523	+0.090	0.076	1.19	0.233
NMR	0.499	+0.114	0.084	1.35	0.176

Comparisons performed by the DeLong method in the 104 patients with all eleven indices and an assigned Mandard grade available, of whom 29 achieved a major pathological response. Because a single complete-case subset is required for correlated ROC comparison, the areas under the curve differ slightly from those in Supplementary Table S1, which uses index-specific denominators. Each index was oriented so that the direction expected to favour major pathological response corresponds to a higher value. PNI did not differ significantly from any other index; the smallest of the ten *p* values was 0.176. Rows are ordered by AUC.

Table S7. Missing baseline data by variable and by institution.

Variable	Centre A (n = 74)	Centre B (n = 56)	Total (n = 130)	*p*
C-reactive protein (determines mGPS)	2 (2.7%)	12 (21.4%)	14 (10.8%)	0.001
Mandard tumour regression grade	7 (9.5%)	0 (0.0%)	7 (5.4%)	0.019
Serum albumin (determines PNI, HALP)	4 (5.4%)	0 (0.0%)	4 (3.1%)	0.134
Total bilirubin (determines ALBI, PALBI)	7 (9.5%)	1 (1.8%)	8 (6.2%)	0.137
CA19-9	3 (4.1%)	4 (7.1%)	7 (5.4%)	0.463
Neutrophil, lymphocyte, monocyte, platelet counts	0 (0.0%)	0 (0.0%)	0 (0.0%)	—

Values are the number (percentage) of patients with the variable missing. Centre A is the Dokuz Eylül University Faculty of Medicine and Centre B is Izmir City Hospital. *p* values are from Fisher's exact test comparing missingness between institutions. Missingness in C-reactive protein and in Mandard grade differed significantly by centre. Patients with and without a C-reactive protein measurement had similar median PNI (50.8 versus 50.4; $p = 0.939$) and CA19-9 (11.5 versus 12.8; $p = 0.782$), but the major pathological response rate differed (28.4% versus 7.1%), indicating that missingness in C-reactive protein is not independent of the outcome.

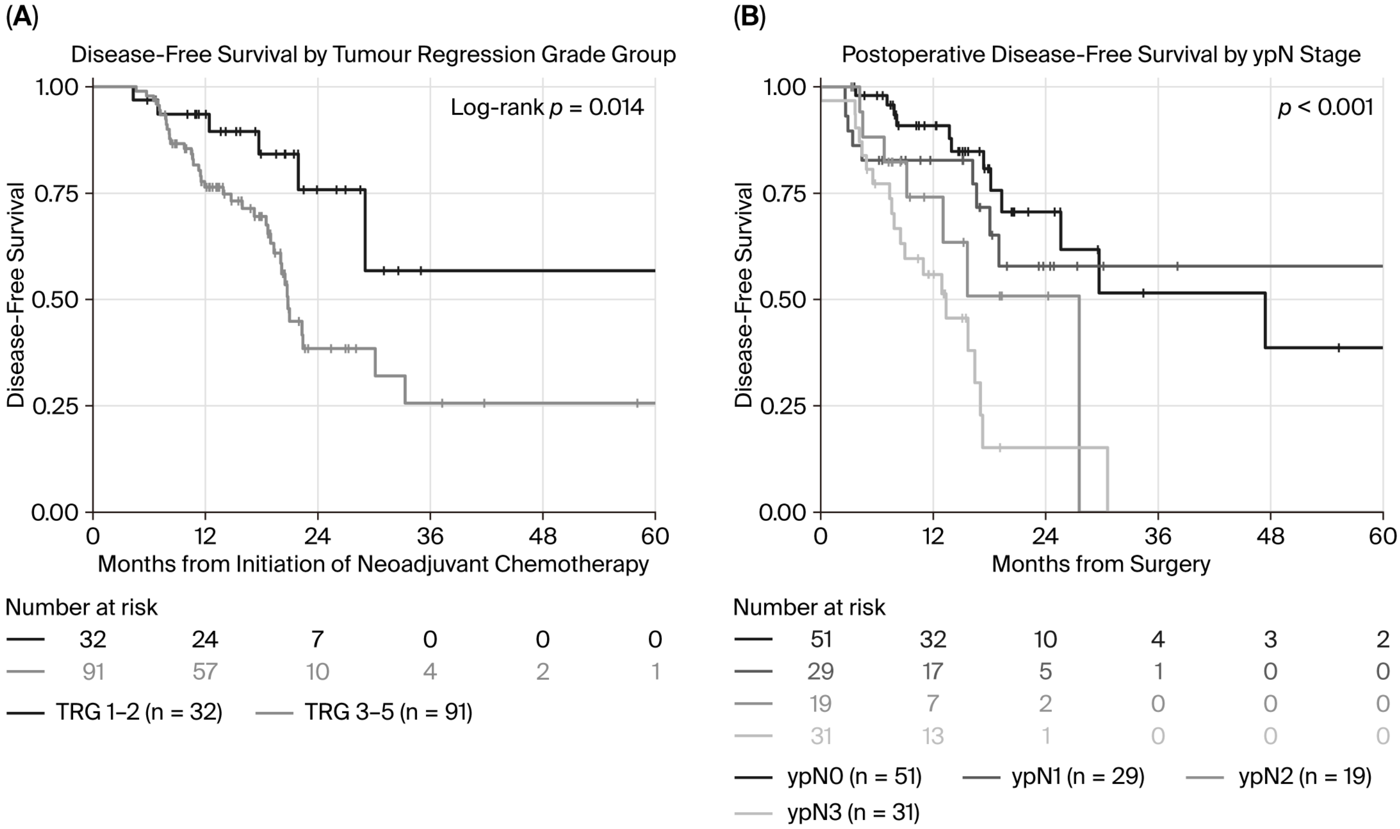

Figure S1. Kaplan–Meier estimates of disease-free survival. (**A**) By Mandard tumor regression grade group, measured from the initiation of neoadjuvant chemotherapy, in the 123 patients with an assigned grade; median disease-free survival was not reached in the TRG 1–2 group and was 20.8 months in the TRG 3–5 group (log-rank $p = 0.014$). (**B**) By post-treatment nodal stage, measured from the date of surgery, in all 130 patients; median disease-free survival was 47.4 months for ypN0, not reached for ypN1, 27.6 months for ypN2 and 13.4 months for ypN3 (log-rank $p < 0.001$). Vertical ticks denote censored observations. Both stratifying variables are pre-specified categorical variables; no data-derived index cut-off was used in any survival analysis.

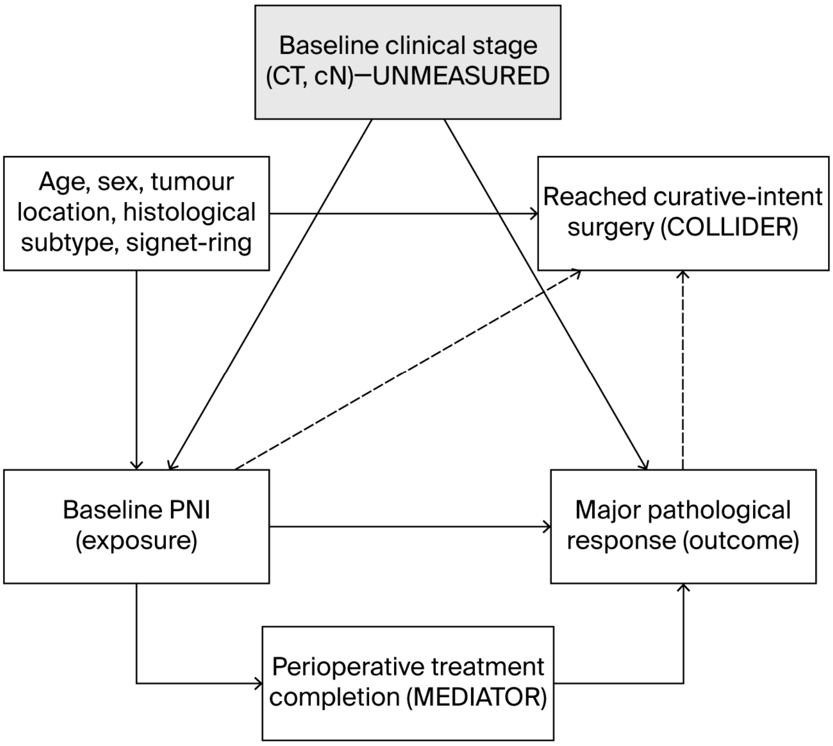

Figure S2. Directed acyclic graph for the association between baseline PNI and major pathological response. Treatment completion lies on the causal path from PNI to response and was therefore not adjusted for. Baseline clinical stage is an unmeasured confounder. The analysis is conditioned on reaching surgery, which is a collider on paths from both PNI and response. Solid arrows: assumed causal effects. Dashed arrows: paths into the selection variable. Shaded node: not measured in this study.

	PNI	NLR	NMR	LMR	PLR	SII	SIRI	HALP	ALBI	PALBI	mGPS
PNI	1.00	−0.58	−0.18	0.52	−0.62	−0.48	−0.37	0.67	−0.70	−0.27	−0.28
NLR	−0.58	1.00	0.55	−0.66	0.58	0.79	0.81	−0.47	0.15	0.05	0.16
NMR	−0.18	0.55	1.00	0.18	0.32	0.54	0.16	−0.27	0.00	0.12	0.02
LMR	0.52	−0.66	0.18	1.00	−0.46	−0.49	−0.84	0.37	−0.19	0.00	−0.21
PLR	−0.62	0.58	0.32	−0.46	1.00	0.79	0.39	−0.83	0.25	0.36	0.20
SII	−0.48	0.79	0.54	−0.49	0.79	1.00	0.71	−0.66	0.25	0.35	0.25
SIRI	−0.37	0.81	0.16	−0.84	0.39	0.71	1.00	−0.30	0.12	0.03	0.22
HALP	0.67	−0.47	−0.27	0.37	−0.83	−0.66	−0.30	1.00	−0.42	−0.38	−0.25
ALBI	−0.70	0.15	0.00	−0.19	0.25	0.25	0.12	−0.42	1.00	0.66	0.26
PALBI	−0.27	0.05	0.12	0.00	0.36	0.35	0.03	−0.38	0.66	1.00	0.11
mGPS	−0.28	0.16	0.02	−0.21	0.20	0.25	0.22	−0.25	0.26	0.11	1.00

Figure S3. Spearman correlation matrix of the eleven indices (n = 130; pairwise complete observations). Darker shading indicates stronger correlation in either direction. Seven pairs reached |rho| ≥ 0.70: LMR-SIRI (−0.84), PLR-HALP (−0.83), NLR-SIRI (+0.81), PLR-SII (+0.79), NLR-SII (+0.79), SII-SIRI (+0.71) and PNI-ALBI (−0.70), reflecting shared leukocyte, platelet and albumin components.
